# Predictive performance of blood urea nitrogen, cystatin C, and their ratio for in-hospital mortality in sepsis: a Chinese two-center analysis

**DOI:** 10.1515/med-2026-1522

**Published:** 2026-10-01

**Authors:** Zhitao Zhong, Zhihua Xie, Shihao Liu, Lukai Lv, Minyan Fan, Kefeng Li, Chao Chen, Guan Wang, Li Ye, Ping Xu

**Affiliations:** Emergency Department, Zigong Fourth People’s Hospital, Zigong, Sichuan, China; Institute of Medical Big Data, Zigong Academy of Artificial Intelligence and Big Data for Medical Science, Zigong, China; Emergency Department, Fushun People’s Hospital, Zigong, Sichuan, China; Faculty of Applied Sciences, Macao Polytechnic University, Macao SAR, China; School of Computer Science and Engineering, Sichuan University of Science and Engineering, Zigong, China; Innovation Center of Nursing Research, Nursing Key Laboratory of Sichuan Province, National Clinical Research Center for Geriatrics, West China Hospital, Sichuan University, Chengdu, Sichuan, China

**Keywords:** sepsis, blood urea nitrogen, cystatin C, blood urea nitrogen-cystatin c ratio, prognosis

## Abstract

**Objectives:**

Early identification of septic patients at high risk of mortality is crucial for improving outcomes. Renal function indicators such as blood urea nitrogen (BUN) and cystatin C (Cys C) have been individually associated with sepsis outcomes. However, the comparative prognostic value of these indicators and their ratio remained unclear.

**Methods:**

We conducted a retrospective cohort study in two hospitals in China. Associations between renal indicators and mortality were examined using multivariable logistic regression, restricted cubic spline modelling, and threshold analysis. Discrimination was assessed with receiver operating characteristic (ROC) curves, net reclassification improvement (NRI), and integrated discrimination improvement (IDI), evaluated both individually and in combination with the SOFA score. Clinical applicability was examined with calibration and decision curve analyses.

**Results:**

A total of 1,039 patients were included in this study. After adjustment for covariates, multivariable logistic regression identified both BCR (OR 1.247, 95 % CI 1.165–1.335, p<0.001) and BUN (OR 1.047, 95 % CI 1.029–1.065, p<0.001) as independent predictors of in-hospital mortality among patients with sepsis, and these findings were replicated in the independent external cohort. In the exploration cohort, both BCR and BUN exhibited nonlinear associations with mortality risk. Incorporating BCR into the SOFA score resulted in the highest overall predictive performance (AUC 0.717) and the greatest net clinical benefit (0.106). Reclassification analyses further demonstrated that BCR achieved higher NRI and IDI values than BUN or Cys C, and that adding BCR to the SOFA score improved both NRI and IDI compared with SOFA alone.

**Conclusions:**

BCR and BUN were independently associated with in-hospital mortality in patients with sepsis. BCR showed modestly better prognostic performance than BUN or Cys C alone, and its integration with SOFA may provide supplementary risk-stratification information. However, BCR should not be considered a replacement for established severity scores, and further prospective validation is warranted.

## Introduction

Microorganisms like bacteria, viruses, fungi, or parasites usually cause infections that trigger sepsis [1], 2].Sepsis is a medical condition characterized by an abnormal host response to infection, potentially leading to multiple organ failure and death [3], [4], [5]. Annually, it is estimated to lead to six million fatalities, making it a leading cause of death in intensive care units worldwide [1]. The increasing number of sepsis-related cases and deaths stresses the urgent demand for improved therapeutic solutions [1], 6]. Early detection of septic patients who are at a higher risk of dying, along with prompt treatment, is crucial for decreasing deaths related to sepsis [7].

Several studies have reported that renal function indicators, such as blood urea nitrogen (BUN) and cystatin C (Cys C), are associated with mortality risk in patients with sepsis. According to a multi-center retrospective study from the eICU Collaborative Research Database v2.0, admission BUN levels independently forecasted 28-day mortality in critically ill septic patients [8]. Additionally, a prospective observational study highlighted that plasma Cys C levels at intensive care unit (ICU) admission were associated with increased 90-day mortality [9].

In critically ill populations, ratios derived from renal-function-related indicators have also been explored for prognostic assessment and may provide additional information beyond single indicators. According to a retrospective study from the Medical Information Mart for Intensive Care IV (MIMIC-IV) version 2.2, the area under the curve (AUC) values indicated that the creatinine-to-albumin ratio was better than creatinine, albumin, and the sequential organ failure assessment (SOFA) score [10]. Furthermore, the urea-creatinine ratio has demonstrated superior predictive performance in various conditions, such as trauma-related acute respiratory distress syndrome and severe burns associated with acute kidney injury, compared to measurements of BUN or creatinine alone [11], 12]. Despite these findings, the comparative predictive value of BUN, Cys C, and the blood urea nitrogen-cystatin c ratio (BCR) for sepsis prognosis remained ambiguous.

Given that BUN and Cys C may reflect different but complementary aspects of renal dysfunction, metabolic burden, and disease severity in sepsis, their ratio may provide additional prognostic information beyond either marker alone. We hypothesized that a higher BCR would be associated with an increased risk of in-hospital mortality and might provide additional prognostic value compared with BUN or Cys C alone. Therefore, this study aimed to compare the associations and predictive performance of BUN, Cys C, and BCR for in-hospital mortality in patients with sepsis and to further evaluate whether BCR could provide additional clinical value when combined with the SOFA score.

## Methods

### Data source

This dual-center study was conducted in China. Data from Zigong Fourth People’s Hospital were derived from an updated version of its historical ICU database [13], covering records of patients with infections admitted between 2018 and 2024. Data from Fushun People’s Hospital encompassed ICU admissions for infectious diseases from 2019 to 2024 and were used as an independent external cohort to examine the reproducibility of the findings.

### Participant selection

The inclusion criteria were as follows: 1) Patients admitted to the central ICU or emergency ICU, and diagnosed with sepsis according to the Sepsis 3.0 guidelines [3]; 2) Individuals aged 18 years or older at the time of ICU admission. The exclusion criteria included: 1) Patients with more than one ICU admission, where only data from the initial admission were used for analysis; 2) Patients lacking essential variable data, such as BUN and Cys C, within the first 24 h of ICU admission.

## Clinical outcomes

The primary endpoint was all-cause mortality during hospitalization. Because the exact cause of death could not be reliably adjudicated from the retrospective electronic medical records, sepsis-specific mortality was not used as the primary outcome.

### Data extraction

The demographics of patients included both age and gender. Vital signs such as heart rate, systolic and diastolic blood pressure (SBP and DBP), temperature, respiratory rate, and pulse oxygen saturation (SpO_2_) were extracted. Extensive lab tests were performed, including BUN, Cys C, creatinine, uric acid (UA), white blood cell count (WBC), lymphocyte count, neutrophil count, monocyte count, hemoglobin (HB), hematocrit (HCT), platelet count (PLT), alanine aminotransferase (ALT), aspartate aminotransferase (AST), direct bilirubin (DBIL), indirect bilirubin (IBIL), albumin, globulin, potassium, sodium, chloride, calcium, D-dimer, prothrombin time (PT), activated partial thromboplastin time (APTT), and fibrinogen (FIB). Comorbidities included congestive heart failure (CHF), chronic obstructive pulmonary disease (COPD), and chronic kidney disease (CKD). During the hospital stay, treatments including mechanical ventilation and continuous renal replacement therapy (CRRT) were analyzed. Additionally, the condition of the patients was also evaluated by assessing the severity of the illness with the SOFA score.

### Statistical analysis

Quantitative data following a normal distribution were shown as mean ± standard deviation (x̄ ± s), whereas data not following a normal distribution were represented by median (interquartile range [IQR]). Categorical variables were detailed using frequencies and percentages. Patients were divided into two categories according to their status during hospitalization: survivors and non-survivors, with descriptive statistics for all variables created using the CBCgrps package [14]. Candidate variables were screened using the Boruta algorithm based on random forest classification (set.seed=123, doTrace=0). The Boruta algorithm was used only to assist in identifying potentially important clinical covariates among the available baseline variables for subsequent multivariable adjustment. Only variables with a variance inflation factor (VIF) less than 5 were included in the multivariate logistic regression analyses [15], 16]. BUN, Cys C, and BCR tertiles were used as categorical variables in various adjusted models to assess odds ratios (ORs) with 95 % confidence intervals (CIs) for in-hospital all-cause mortality. Restricted cubic spline (RCS) analysis was applied to identify possible nonlinear relationships between exposure variables and outcomes. Receiver operating characteristic (ROC) curves were created for BCR, BUN, and Cys C, both individually and in combination with the SOFA score, to evaluate their predictive accuracy for in-hospital mortality. The improvement in predicting risk for renal function indicators was measured by the net reclassification improvement (NRI) and integrated discrimination improvement (IDI) indices [17]. Calibration curves and decision curve analysis (DCA) were employed to evaluate the clinical applicability. Missing data were handled using multiple imputation by chained equations with the mice package in R. The proportions of missing values are presented in Table S1. Statistical analyses were performed with R version 4.5.2, and a significance level of p<0.05 was applied to all tests.

## Ethical approval

Due to the retrospective design of the study and the fact that all patient data were de-identified before analysis, the Ethics Committee granted a waiver of informed consent. All procedures adhered to the ethical principles of the hospital and patient data privacy was rigorously protected. Ethical Approval was obtained from the Ethics Committee of Zigong Fourth People’s Hospital (Approval No. 2025-033) and the Ethics Committee of Fushun People’s Hospital (Approval No. 2025-03).

## Results

### Patient demographics and baseline characteristics

A total of 1,039 patients with a diagnosis of sepsis were analyzed, comprising 634 cases in the exploration cohort and 405 in the independent external cohort (Figure S1). The respective in-hospital mortality rates were 34.5 % and 16.5 %, and in both cohorts the majority of patients were male (Table 1 and Table S2). The median age was 74 years (IQR 66–83) in the exploration cohort and 73 years (IQR 65–81) in the independent external cohort, indicating that the study population mainly consisted of older adults. In the exploration cohort, non-survivors tended to have higher heart rates, lower DBP, and a greater prevalence of CHF and CKD compared with survivors. Across both cohorts, BUN levels and the BCR were elevated among non-survivors (p<0.05). Disease severity, assessed by the SOFA score, was consistently greater in non-survivors from both cohorts.

**Table 1:** Baseline characteristics of sepsis patients in the exploration cohort.

Variables	Overall (n=634)	Survivors (n=415)	Non-survivors (n=219)	p-Value
**Demographics**				

Male, %	416 (65.62)	273 (65.78)	143 (65.30)	0.902
Age, years, median (IQR)	74.0 (66.0, 83.0)	74.0 (67.0, 82.0)	75.0 (66.0, 84.5)	0.332

**Vital signs**				

Temperature, °C, median (IQR)	36.4 (36.1, 36.7)	36.4 (36.1, 36.7)	36.4 (36.1, 36.7)	0.897
Heart rate, bpm, median (IQR)	125.0 (107.0, 142.8)	124.0 (106.5, 139.0)	131.0 (108.0, 153.5)	**0.003**
Respiratory rate, bpm, median (IQR)	27.0 (17.0, 34.0)	27.0 (17.0, 33.0)	28.0 (17.5, 35.0)	0.228
SBP, mmHg, median (IQR)	111.0 (91.0, 133.0)	113.0 (92.0, 134.5)	108.0 (88.5, 128.0)	0.055
DBP, mmHg, median (IQR)	64.0 (53.0, 78.0)	65.0 (54.0, 80.5)	63.0 (50.0, 73.0)	**0.005**
SpO_2_, %, median (IQR)	96.0 (92.0, 99.0)	96.0 (93.0, 98.0)	96.0 (92.0, 99.0)	0.884

**Comorbidities**				

CHF, %	257 (40.54)	147 (35.42)	110 (50.23)	**<0.001**
COPD, %	176 (27.76)	124 (29.88)	52 (23.74)	0.101
CKD, %	186 (29.34)	103 (24.82)	83 (37.90)	**<0.001**

**Laboratory tests**				

BCR	5.60 (4.12, 7.20)	5.12 (3.62, 6.65)	6.29 (5.07, 7.97)	**<0.001**
BUN, mmol/L, median (IQR)	10.89 (7.13, 18.05)	9.74 (6.64, 15.20)	14.39 (9.34, 22.16)	**<0.001**
Cys C, mg/L, median (IQR)	2.08 (1.38, 3.25)	2.02 (1.38, 3.21)	2.20 (1.38, 3.31)	0.608
Creatinine, umol/L, median (IQR)	118.25 (73.58, 199.50)	109.40 (72.35, 187.95)	127.30 (76.80, 211.85)	0.082
UA, umol/L, median (IQR)	321.00 (93.09, 518.00)	296.00 (92.43, 495.00)	373.00 (100.54, 564.00)	**0.037**
WBC, 10^9^/L, median (IQR)	10.63 (6.62, 16.98)	10.72 (6.79, 16.77)	10.40 (6.29, 17.45)	0.737
Lymphocyte count, 10^9^/L, median (IQR)	0.56 (0.33, 0.95)	0.55 (0.33, 0.96)	0.59 (0.34, 0.95)	0.747
Neutrophil count, 10^9^/L, median (IQR)	10.12 (6.26, 15.72)	10.16 (6.62, 15.32)	9.81 (5.85, 16.35)	0.579
Monocyte count, 10^9^/L, median (IQR)	0.41 (0.21, 0.71)	0.42 (0.21, 0.70)	0.39 (0.18, 0.70)	0.328
HB, g/L, mean ± SD	110.14 ± 27.55	110.25 ± 27.74	109.93 ± 27.26	0.891
HCT, L/L, mean ± SD	0.34 ± 0.08	0.34 ± 0.08	0.34 ± 0.08	0.971
PLT, 10^9^/L, median (IQR)	162.0 (101.3, 241.8)	164.0 (110.0, 243.0)	153.0 (87.5, 238.5)	0.079
ALT, U/L, median (IQR)	26.95 (16.00, 52.60)	26.40 (15.45, 51.00)	28.40 (17.35, 61.00)	0.34
AST, U/L, median (IQR)	41.85 (24.80, 89.82)	40.70 (24.15, 80.95)	45.10 (25.40, 99.30)	0.195
DBIL, umol/L, median (IQR)	6.00 (3.50, 11.17)	5.70 (3.30, 10.75)	6.40 (3.85, 12.30)	0.122
IBIL, umol/L, median (IQR)	7.30 (4.70, 11.90)	7.20 (4.65, 11.40)	7.50 (4.80, 12.95)	0.488
Albumin, g/L, mean ± SD	29.95 ± 6.41	29.91 ± 6.23	30.02 ± 6.75	0.838
Globulin, g/L, median (IQR)	28.50 (24.63, 32.80)	28.30 (24.30, 32.40)	29.10 (25.00, 33.05)	0.365
Potassium, mmol/L, median (IQR)	3.93 (3.40, 4.50)	3.92 (3.40, 4.46)	3.98 (3.41, 4.51)	0.376
Sodium, mmol/L, median (IQR)	138.0 (133.6, 142.2)	137.8 (133.5, 141.7)	138.3 (133.6, 143.9)	0.195
Chloride, mmol/L, median (IQR)	101.3 (96.2, 106.0)	101.1 (96.2, 105.9)	101.5 (96.2, 106.1)	0.671
Calcium, mmol/L, median (IQR)	2.15 (2.03, 2.29)	2.14 (2.03, 2.27)	2.17 (2.05, 2.34)	0.050
D-dimer, mg/L, median (IQR)	4.88 (2.10, 10.59)	4.72 (1.98, 10.22)	5.44 (2.37, 11.53)	0.25
PT, sec, median (IQR)	14.20 (12.80, 15.90)	14.10 (12.70, 15.90)	14.40 (13.00, 15.95)	0.187
APTT, sec, median (IQR)	30.80 (26.90, 36.05)	30.80 (26.85, 35.10)	31.20 (27.05, 37.70)	0.16
FIB, g/L, median (IQR)	4.16 (3.00, 5.60)	4.17 (2.98, 5.62)	4.16 (3.09, 5.60)	0.764

**Treatment during hospitalization**				

CRRT, %	59 (9.31)	33 (7.95)	26 (11.87)	0.106
Ventilator, %	562 (88.64)	352 (84.82)	210 (95.89)	**<0.001**
**SOFA score**	8.00 (6.00, 10.00)	7.00 (6.00, 9.00)	9.00 (7.00, 11.00)	**<0.001**

Continuous variables are expressed as mean ± SD, or Median (IQR), categorical variables are expressed as number (%). IQR, interquartile range; SD, standard deviation; SBP, systolic blood pressure; DBP, diastolic blood pressure; SpO2, pulse oxygen saturation; CHF, congestive heart failure; COPD, chronic obstructive pulmonary disease; CKD, chronic kidney disease; BUN, blood urea nitrogen; Cys C, cystatin C; BCR, blood urea nitrogen-cystatin c ratio; UA, uric acid; WBC, white blood cell count; HB, hemoglobin; HCT, hematocrit; PLT, platelet; PT, prothrombin time; APTT, activated partial thromboplastin time; FIB, fibrinogen; ALT, alanine aminotransferase; AST, aspartate aminotransferase; DBIL, direct bilirubin; IBIL, indirect bilirubin; SOFA, sequential organ failure assessment; CRRT, continuous renal replacement therapy. Bold values indicate statistical significance at p<0.05.

### Association between the BCR and in-hospital mortality

The Boruta algorithm identified ventilator use, CHF, heart rate, and platelet count as key variables (Figure 1). Age and gender were added as demographic covariates. No evidence of multicollinearity was observed, with all VIF below 5 (Table S3). Sequential multivariable logistic regression models were then developed incorporating these factors. In the exploration cohort, after full adjustment, both the BCR (OR 1.247, 95 % CI 1.165–1.335, p<0.001) and BUN (OR 1.047, 95 % CI 1.029–1.065, p<0.001) remained independent predictors of in-hospital mortality among patients with sepsis, whereas Cys C was not statistically associated with outcome (Table 2). Findings from the independent external cohort were in line with these results, showing similar associations for BCR and BUN in the independent external cohort (Table S4). When grouped by tertiles, mortality risk rose progressively across increasing categories of BCR and BUN (p for trend <0.001). Patients in the highest tertile (T3) faced the greatest risk compared with those in the lowest tertile (T1).

**Figure 1:**
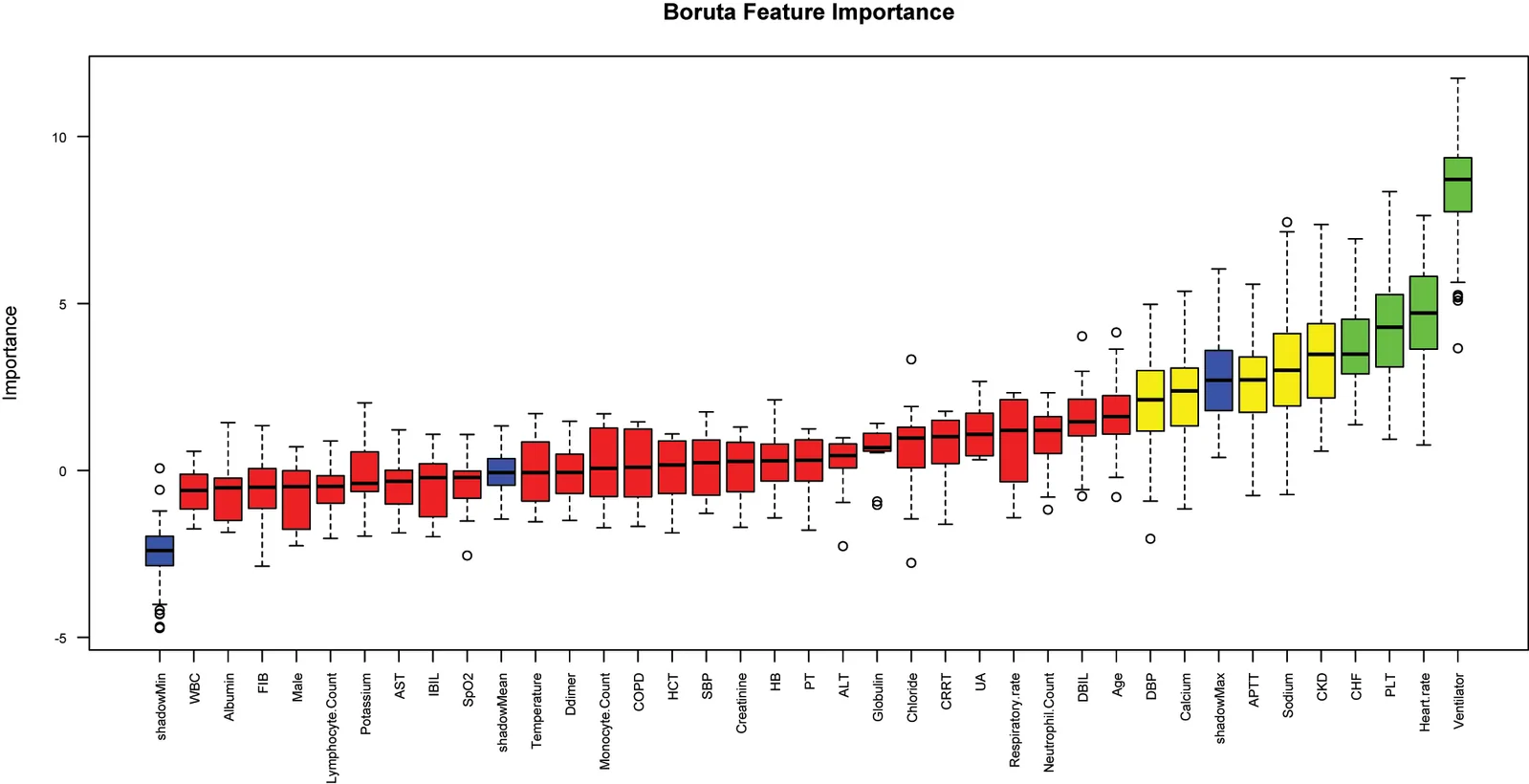
Results of covariate selection using the boruta algorithm. Note: Because the SOFA score comprises multiple correlated components, the SOFA score was excluded from the Boruta feature-selection process. BUN, Cys C, and BCR, as the exposure variables of interest, were also excluded from covariate selection. SBP, systolic blood pressure; DBP, diastolic blood pressure; SpO 2 , pulse oxygen saturation; CHF, congestive heart failure; COPD, chronic obstructive pulmonary disease; CKD, chronic kidney disease; BUN, blood urea nitrogen; Cys C, cystatin C; BCR, blood urea nitrogen-cystatin c ratio; UA, uric acid; WBC, white blood cell count; HB, hemoglobin; HCT, hematocrit; PLT, platelet; PT, prothrombin time; APTT, activated partial thromboplastin time; FIB, fibrinogen; ALT, alanine aminotransferase; AST, aspartate aminotransferase; DBIL, direct bilirubin; IBIL, indirect bilirubin; SOFA, sequential organ failure assessment; CRRT, continuous renal replacement therapy.

**Table 2:** Multimodel logistic regression analysis of predictors associated with in-hospital mortality among sepsis patients in the exploration cohort.

Variables	Model1		Model2		Model3	
	OR (95 % CI)	p-Value	OR (95 % CI)	p-Value	OR (95 % CI)	p-Value
BCR	1.228 (1.148–1.312)	**<0.001**	1.228 (1.149–1.313)	**<0.001**	1.247 (1.165–1.335)	**<0.001**
T1	1.000 (reference)		1.000 (reference)		1.000 (reference)	
T2	2.036 (1.319–3.143)	**0.001**	2.026 (1.312–3.129)	**0.001**	2.200 (1.406–3.442)	**<0.001**
T3	3.375 (2.197–5.185)	**<0.001**	3.377 (2.197–5.190)	**<0.001**	3.665 (2.350–5.716)	**<0.001**
P for trend		**<0.001**		**<0.001**		**<0.001**
BUN	1.040 (1.023–1.057)	**<0.001**	1.040 (1.023–1.057)	**<0.001**	1.047 (1.029–1.065)	**<0.001**
T1	1.000 (reference)		1.000 (reference)		1.000 (reference)	
T2	2.072 (1.335–3.217)	**0.001**	2.051 (1.313–3.203)	**0.002**	2.026 (1.280–3.207)	**0.003**
T3	3.864 (2.504–5.961)	**<0.001**	3.835 (2.479–5.933)	**<0.001**	4.314 (2.738–6.798)	**<0.001**
P for trend		**<0.001**		**<0.001**		**<0.001**
Cys C	1.028 (0.932–1.134)	0.575	1.027 (0.931–1.133)	0.600	1.030 (0.929–1.143)	0.575
T1	1.000 (reference)		1.000 (reference)		1.000 (reference)	
T2	1.059 (0.707–1.585)	0.783	1.027 (0.682–1.546)	0.900	0.972 (0.635–1.486)	0.894
T3	1.235 (0.825–1.847)	0.305	1.203 (0.801–1.807)	0.372	1.240 (0.810–1.898)	0.322
P for trend		0.287		0.334		0.250

Model 1 adjusted for: none; Model 2 adjusted for: age and gender; Model 3 adjusted for: Model 2 + ventilator, congestive heart failure, heart rate, and platelet count. T, tertiles; OR, odds ratios; CI, confidence interval; BUN, blood urea nitrogen; Cys C, cystatin C; BCR, blood urea nitrogen-cystatin c ratio. Bold values indicate statistical significance at p<0.05.

### Restricted cubic spline and threshold analyses

RCS modeling suggested nonlinear associations of BCR and BUN with in-hospital mortality in the exploration cohort (p for non-linearity <0.05) (Figure 2). Threshold analysis further identified distinct inflection points for both markers (p for likelihood ratio test <0.001) (Table 3). A BCR value ≥2.607 corresponded to a sharp increase in mortality risk (OR 1.170, 95 % CI 1.094–1.252, p<0.001), while a BUN level ≤20.66 mmol/L was similarly associated with elevated risk (OR 1.104, 95 % CI 1.058–1.152, p<0.001). In the independent external cohort, RCS indicated approximately linear positive associations between BCR, BUN, and in-hospital mortality (p for nonlinearity >0.05) (Figure S2).

**Figure 2:**
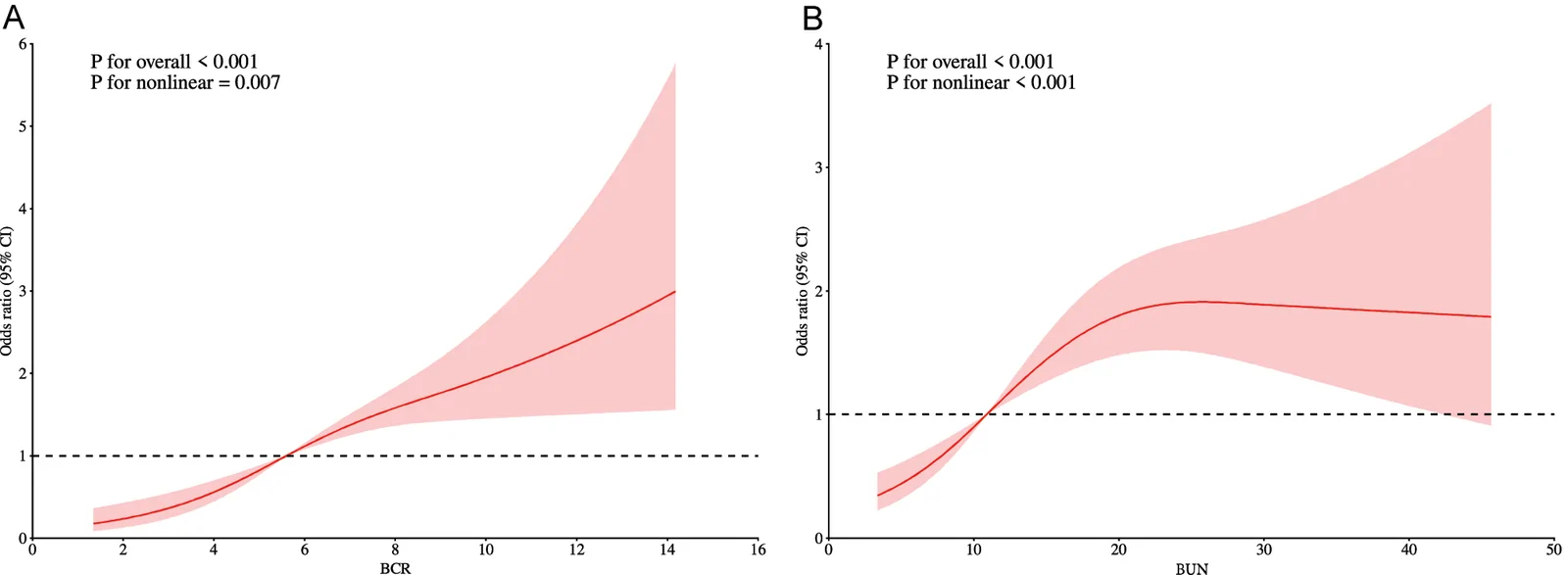
Restricted cubic spline analysis in the exploration cohort. Abbreviations: OR, odds ratios; CI, confidence interval; BUN, blood urea nitrogen; BCR, blood urea nitrogen-cystatin c ratio.

**Table 3:** Threshold analysis of BCR and BUN for in-hospital mortality in the exploration cohort.

Variable	Threshold	Category	OR (95 % CI)	p-Value	P For likelihood ratio test
BCR	2.607	<2.607	1.000 (0.000–Inf)	1	**<0.001**
		≥2.607	1.170 (1.094–1.252)	**<0.001**	
BUN, mmol/L	20.66	<20.66	1.104 (1.058–1.152)	**<0.001**	**<0.001**
		≥20.66	0.984 (0.947–1.023)	0.422	

OR, odds ratios; CI, confidence interval; BUN, blood urea nitrogen; BCR, blood urea nitrogen-cystatin c ratio. Bold values indicate statistical significance at p<0.05.

### Predictive performance of different variables and their combinations

Among the three biomarkers evaluated (BCR, BUN, and Cys C), BCR showed the largest AUC in the exploration cohort (0.659, 95 % CI 0.616–0.703). The same pattern was observed in the independent external cohort, where BCR again achieved the highest AUC (0.619, 95 % CI, 0.548–0.691) (Figure 3). When each biomarker was combined with the SOFA score, the SOFA + BCR model showed the highest AUC among the evaluated models, yielding an AUC of 0.717 (95 % CI 0.675–0.759) in the exploration cohort and 0.702 (95 % CI 0.635–0.769) in the independent external cohort. In the exploration cohort, BCR showed greater NRI and IDI than BUN or Cys C, and adding BCR to the SOFA score improved NRI and IDI compared with SOFA alone (Table 4). However, this incremental gain was not fully reproduced in the independent external cohort (Table S5).

**Figure 3:**
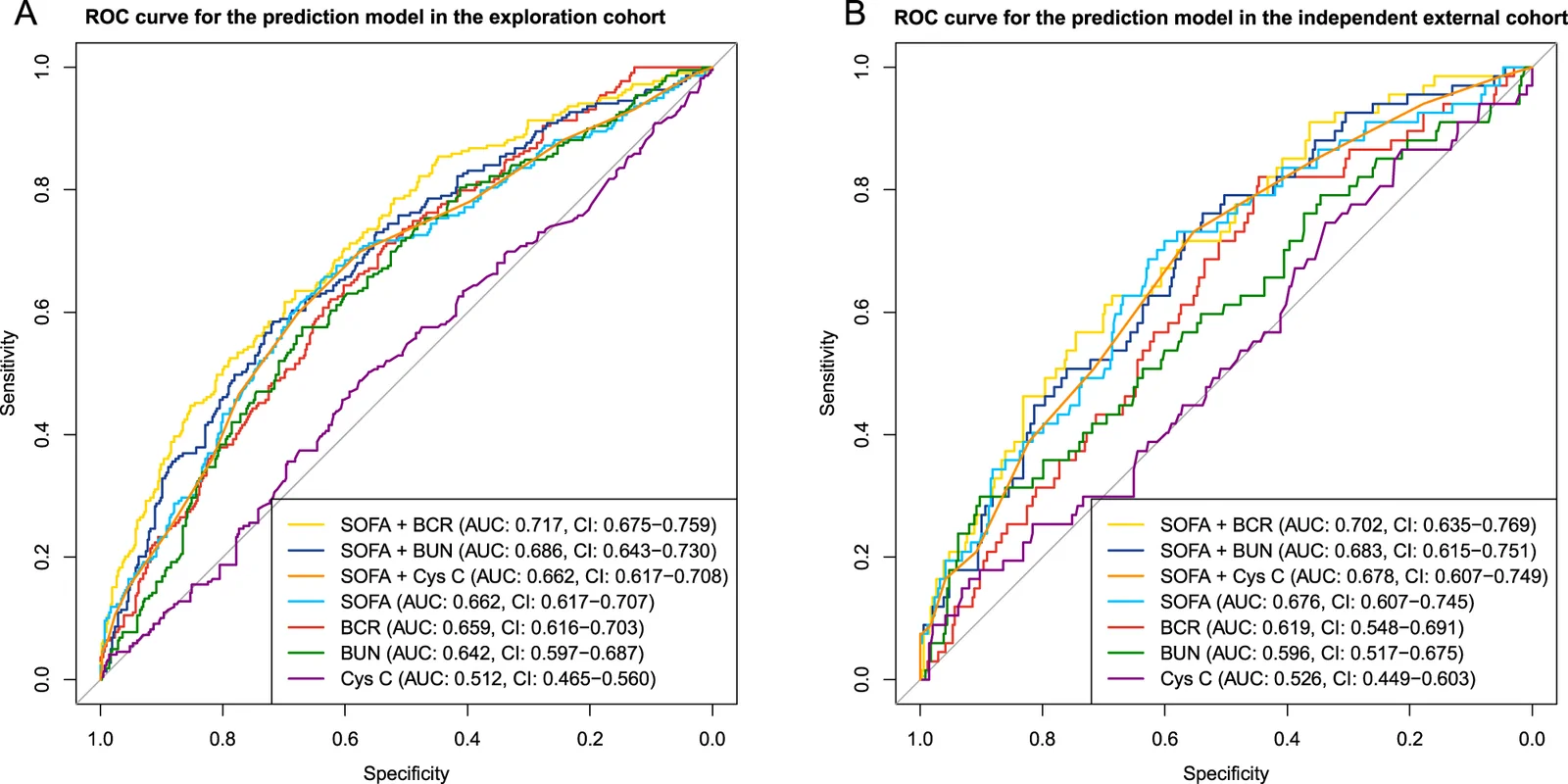
Receiver operating characteristic curve analysis in the two cohorts. Abbreviations: AUC, area under the curve; CI, confidence interval; SOFA, sequential organ failure assessment; BUN, blood urea nitrogen; Cys C, cystatin C; BCR, blood urea nitrogen-cystatin c ratio.

**Table 4:** NRI and IDI analysis in the exploration cohort.

Variables	NRI (95 % CI)	p-Value	IDI (95 % CI)	p-Value
BCR + SOFA vs. SOFA	0.394 (0.234–0.555)	**<0.001**	0.063 (0.042–0.084)	**<0.001**
BUN + SOFA vs. SOFA	0.377 (0.220–0.535)	**<0.001**	0.017 (0.006–0.028)	**0.002**
Cys C + SOFA vs. SOFA	−0.049 (−0.207–0.110)	0.549	0.002 (−0.001–0.005)	0.204
BCR vs. BUN	0.184 (0.021–0.347)	**0.026**	0.032 (0.011–0.053)	**0.002**
BCR vs. Cys C	0.403 (0.243–0.564)	**<0.001**	0.069 (0.047–0.092)	**<0.001**

NRI, net reclassification improvement; IDI, integrated discrimination improvement; SOFA, sequential organ failure assessment; CI, confidence interval; BUN, blood urea nitrogen; Cys C, cystatin C; BCR, blood urea nitrogen-cystatin c ratio. Bold values indicate statistical significance at p<0.05.

### Clinical applicability of different variables and their combinations

In both the exploration and independent external cohorts, calibration curves demonstrated close agreement between predicted and observed outcomes for the BCR model and for the combination of BCR with the SOFA score (Hosmer–Lemeshow test p>0.05) (Figure 4 and Figure S3). Decision curve analysis suggested that BCR alone provided a positive net clinical benefit, and the SOFA + BCR model showed the highest net benefit among the evaluated models across part of the threshold-probability range (Figure 4 and Figure S3).

**Figure 4:**
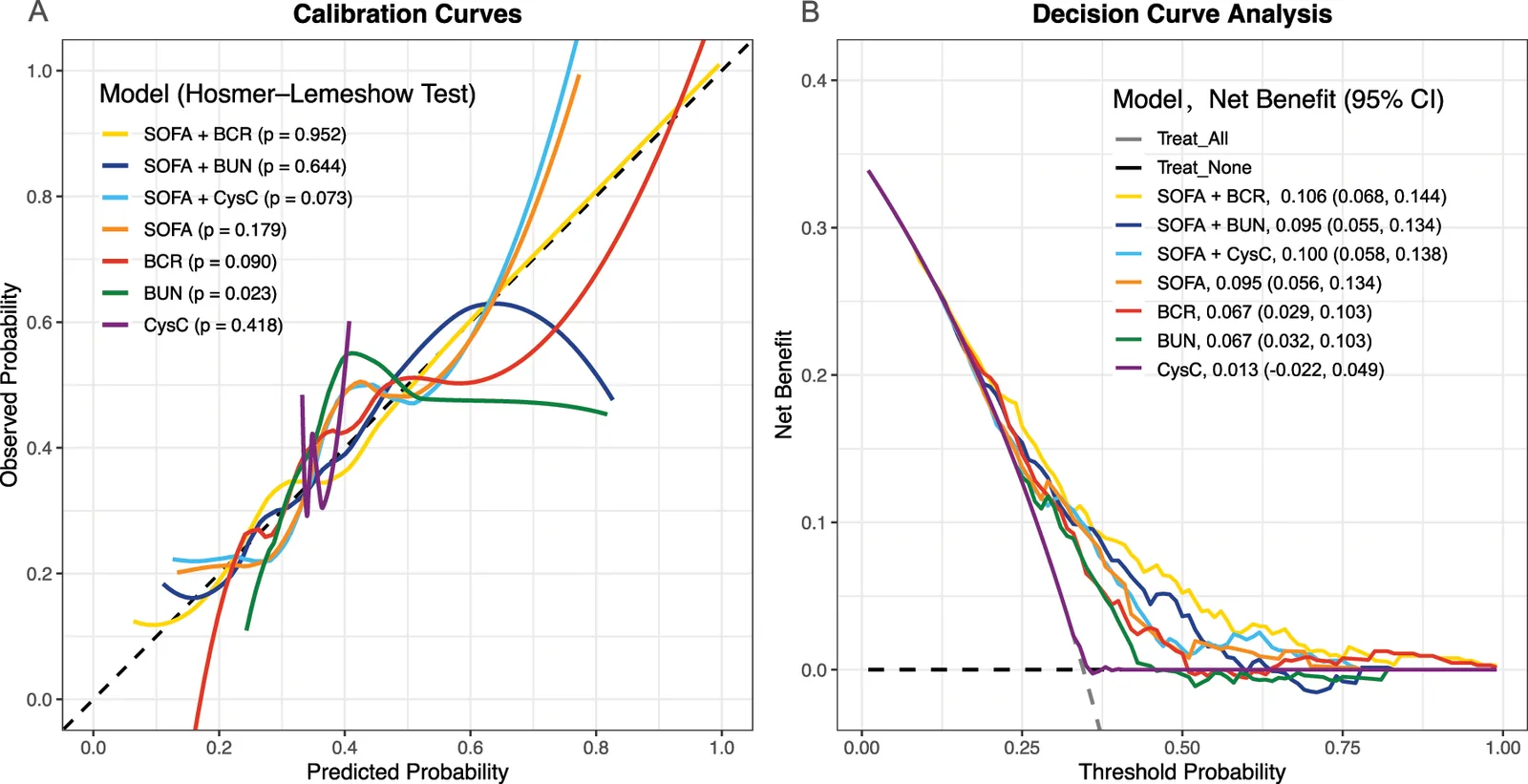
Calibration curve and decision curve analysis in the exploration cohort. Abbreviations: OR, odds ratios; CI, confidence interval; SOFA, sequential organ failure assessment; BUN, blood urea nitrogen; Cys C, cystatin C; BCR, blood urea nitrogen-cystatin c ratio.

## Discussion

In the present study, we found that BCR and BUN measured within 24 h after ICU admission were independently associated with in-hospital mortality in patients with sepsis in both the exploration and independent external cohorts, whereas Cys C alone showed limited predictive performance. In the exploration cohort, the optimal cutoff values were 2.607 for BCR and 20.66 mmol/L for BUN. Compared with BUN and Cys C alone, BCR showed relatively better prognostic performance, and the combination of BCR with the SOFA score provided the highest net clinical benefit. These findings suggest that BCR may serve as a convenient supplementary marker for early risk stratification in patients with sepsis, although it should not be regarded as a replacement for established severity scores such as SOFA.

As a byproduct of protein and amino acid breakdown [18], BUN levels are impacted by different elements, including kidney health and metabolic status [19], 20]. Various studies have established a correlation between BUN and sepsis outcomes. A retrospective study involving 8,817 sepsis patients used SHAP values to determine feature importance, showing that BUN was linked to mortality [21]. On the other hand, a different retrospective study with 12,713 sepsis patients identified a nonlinear association between BUN and 30-day mortality through multivariate Cox regression and smoothed curve fitting [22]. Similar to previous research, we observed an association between BUN and in-hospital mortality in sepsis. In the exploration cohort, RCS analysis suggested a nonlinear pattern, with the association attenuating at higher BUN levels.

Produced by all nucleated cells in the human body [23], Cys C in serum is an effective marker of kidney function and correlates more strongly with direct glomerular filtration rate (GFR) measurements than creatinine [24], 25]. Various studies have highlighted the link between Cys C and sepsis outcomes. A prospective observational study indicated that higher plasma Cys C levels at the time of ICU admission were significantly related to an increased risk of mortality within 90 days [9]. In addition, a study involving 108 ICU patients with sepsis showed that cystatin C had an AUC of 0.765 (95 % CI: 0.663–0.848) for predicting death within 30 days [26]. The differences between our findings and previous studies may be partly explained by the biological scope of Cys C. Cys C mainly reflects glomerular filtration, whereas sepsis-related mortality is driven by systemic inflammation, hemodynamic instability, metabolic stress, and multiorgan dysfunction [3], 27]. Therefore, compared with Cys C alone, BCR may provide broader prognostic information by integrating renal filtration impairment with BUN-related catabolic and metabolic burden in critically ill patients with sepsis.

BCR was identified as an independent risk factor for in-hospital mortality and provided additional prognostic information beyond single indicators. Several mechanisms may explain this finding. Sepsis is commonly characterized by systemic inflammation, hypermetabolism, enhanced protein catabolism, renal hypoperfusion, and acute kidney injury, all of which may contribute to increased BUN levels [28], 29]. In contrast, Cys C mainly reflects glomerular filtration and is less dependent on muscle mass than creatinine [27]. Therefore, an elevated BCR may indicate a disproportionate increase in urea generation or reabsorption relative to filtration impairment, reflecting the combined burden of metabolic stress and renal dysfunction. The BCR cutoff value of 2.607 may provide an exploratory tool for early risk stratification in patients with sepsis. Patients with BCR values above this threshold may require closer monitoring and more comprehensive assessment of organ dysfunction, renal perfusion, metabolic stress, and overall disease severity.

However, BCR should be interpreted as an integrated clinical index rather than a marker of a single biological pathway, because both BUN and Cys C can be influenced by renal function, inflammatory status, fluid balance, medication exposure, and disease severity. Sepsis-related acute kidney injury can directly reduce GFR, leading to the accumulation of both Cys C and BUN [30], 31]. By combining these two biomarkers, BCR may capture subtle changes in renal function while also reflecting the severity of metabolic imbalance, thereby providing a more comprehensive assessment of the pathophysiological status of septic patients. Our findings suggest that BCR, as a combined marker of metabolic and renal dysfunction, may complement existing sepsis prognostic tools. For example, the addition of BCR to the SOFA score improved predictive accuracy for in-hospital mortality. However, because SOFA is a comprehensive organ dysfunction score and the incremental improvement of BCR was modest and not fully reproduced in the independent external cohort, this finding should be interpreted cautiously. Notable discrepancies were found between the exploration and independent external cohorts, particularly in the RCS curve analyses. One possible explanation is that the exploration cohort had a higher in-hospital mortality rate (34.5 %) and elevated SOFA scores (median 8 [6–10]), indicating a generally more severe clinical status. These variations underscore the heterogeneity of sepsis populations and provide additional perspective on the prognostic relevance of renal biomarkers in this context.

Several limitations should also be considered. First, this study evaluated BUN, Cys C, and BCR using laboratory measurements obtained within the first 24 h after ICU admission. Therefore, the present findings mainly reflect the value of these indicators for early risk stratification and cannot capture their dynamic changes during hospitalization or their relationship with treatment response. Future studies incorporating repeated measurements are needed to determine whether longitudinal changes in BUN, Cys C, and BCR provide additional prognostic information. Second, the primary outcome was all-cause in-hospital mortality. Although sepsis-specific mortality or mortality due to sepsis-related complications would be clinically informative, the exact cause of death could not be reliably adjudicated in this retrospective study. In addition, although data were derived from two Chinese medical centers, participants did not include individuals of other ethnicities, which may limit generalizability. Finally, given the retrospective design, bias related to patient selection and data collection cannot be entirely ruled out.

## Conclusions

BUN and BCR were independently associated with in-hospital mortality in patients with sepsis, and BCR showed modestly better prognostic performance than BUN or Cys C alone. Adding BCR to the SOFA score may provide supplementary prognostic information, but BCR should not be considered a substitute for established severity scores. Further studies incorporating dynamic measurements and larger multicenter cohorts are needed to confirm these findings.

## Supplementary Material

Supplementary Material
