# Supplementary material for: Predictive performance of blood urea nitrogen, cystatin C, and their ratio for in-hospital mortality in sepsis: a Chinese two-center analysis
Source: Open Med (Wars). 2026 Oct 1;21(1):20261522. doi: 10.1515/med-2026-1522 (PMC13629720; doi:10.1515/med-2026-1522)
Supplement: Supplementary file 1 [file j_med-2026-1522_suppl_001.docx]

**Table S1.** Missing data for candidate variables in the two cohorts

**Table S2.** Baseline characteristics of sepsis patients in the independent external cohort

**Table S3.** Variance inflation factors among covariates

**Table S4.** Multimodel logistic regression analysis of predictors associated with in-hospital mortality among sepsis patients in the independent external cohort

**Table S5.** NRI and IDI analysis in the independent external cohort
